# Safety of Intravenous Immunoglobulin (Tegeline®), Administered at Home in Patients with Autoimmune Disease: Results of a French Study

**DOI:** 10.1155/2018/8147251

**Published:** 2018-03-15

**Authors:** Eric Hachulla, Gwendal Le Masson, Guilhem Solé, Mohamed Hamidou, Claude Desnuelle, Jean-Philippe Azulay, Gérard Besson, Laure Swiader, Sébastien Abad, Jean-Christophe Antoine, Françoise Bouhour, Alain Créange, Marike Grenouillet, Laurent Magy, Sébastien Marcel, Jean-Michel Paquet, François Rouhart, François Ziegler, Stéphane Mathis, Marc Gauthier-Darnis, Sophie Puget

**Affiliations:** ^1^Department of Internal Medicine, CHU Lille, Lille Nord-de-France University Hospital, Lille, France; ^2^Department of Neurology, Nerve-Muscle Unit, CHU Bordeaux, Pellegrin University Hospital, Bordeaux, France; ^3^Department of Internal Medicine, CHU Nantes, Hôtel Dieu University Hospital, Nantes, France; ^4^Department of Neurology, CHU Nice, L'Archet University Hospital, Nice, France; ^5^Department of Neurology, CHU Marseille, La Timone University Hospital, Marseille, France; ^6^Department of Neurology, CHU Grenoble, Grenoble Alpes University Hospital, Grenoble, France; ^7^Department of Internal Medicine, CHU Marseille, La Timone Hospital University, Marseille, France; ^8^Department of Internal Medicine, Avicenne Hospital, Bobigny, France; ^9^Department of Neurology, CHU Saint-Étienne, Bellevue University Hospital, Saint-Étienne, France; ^10^Department of Neuro-Muscular Diseases, CHU Lyon, Pierre Wertheimer University Hospital, Bron, France; ^11^Department of Neurology, CHU Créteil, Henri Mondor University Hospital, Créteil, France; ^12^Department of Internal Medicine, CHU Bordeaux, Haut-Lévêque University Hospital, Bordeaux, France; ^13^Department of Neurology, CHU Limoges, Dupuytren University Hospital, Limoges, France; ^14^Department of Neurology, Métropole Savoie Hospital, Chambéry, France; ^15^Department of Neurology, Laval Hospital, Laval, France; ^16^Department of Neurology, CHU Brest, University Hospital La Cavale Blanche, Brest, France; ^17^Department of Neurology, Belfort Hospital, Belfort, France; ^18^Market Access & Pricing Department, LFB Biomedicaments, Les Ulis, France; ^19^International Affairs Scientific Unit, LFB Biomedicaments, Les Ulis, France

## Abstract

The efficacy of intravenous immunoglobulins (IVIg) in patients with autoimmune diseases (AID) has been known for several decades. Majority of these patients received IVIg in hospital. A retrospective study was conducted in 22 centers in France to evaluate the feasibility of the administration of Tegeline, an IVIg from LFB Biomedicaments, and assess its safety at home, compared to in hospital, in patients with AID. The included patients were at least 18 years old, suffering from AID, and treated with at least 1 cycle of Tegeline at home after receiving 3 consecutive cycles of hospital-based treatment with Tegeline at a dose between 1 and 2 g/kg/cycle. Forty-six patients with AID, in most cases immune-mediated neuropathies, received a total of 138 cycles of Tegeline in hospital and then 323 at home. Forty-five drug-related adverse events occurred in 17 patients who received their cycles at home compared to 24 adverse events in hospital in 15 patients. Serious adverse events occurred in 3 patients during home treatment, but they were not life-threatening and did not lead to discontinuation of Tegeline. Forty-five patients continued their treatment with Tegeline at home or in hospital; 39 (84.8%) were still receiving home treatment at the end of the study. In conclusion, the study demonstrates the good safety profile of Tegeline administered at home at high doses in patients with AID who are eligible for home administration of Tegeline.

## 1. Introduction

The beneficial effects of intravenous immunoglobulin (IVIg) in patients with autoimmune diseases (AID) were reported as early as 1981 in immune thrombocytopenic purpura (ITP) [1]. The exact mechanism of IVIg in AID is not yet totally understood; however, at doses ranging from 1 to 2 g/kg/cycle, IVIg interfere with both the innate and the adaptive immune systems [2]. IVIg became the main initial and maintenance treatment of demyelinating peripheral neuropathy such as chronic inflammatory demyelinating polyradiculoneuropathy (CIDP) and multifocal motor neuropathy (MMN) with a high and regular dose of 1-2 g/kg/cycle [3, 4]. For other AID (i.e., polymyositis, dermatomyositis, etc.), a high dose of IVIg can be indicated if these patients are refractory to corticosteroids and/or immunosuppressants [5, 6].

Firstly, home-based IVIg for the treatment of primary immunodeficiency (PID) or secondary immunodeficiency (SID) with a low dose (0.4 g/kg/cycle) has been used since the 1990s in Europe and North America [7, 8]. During the same period, home infusion was not yet a widespread practice in patients with AID in France and in many other European countries, probably due to the lack of experience among hospital practitioners and/or the fear of adverse events (AEs). In fact, even if IVIg are generally safe, serious adverse events (SAEs) such as thromboembolic events [9, 10] or renal failure [11] can occur especially in patients with neuromuscular diseases, treated with a high dose of IVIg or associated with concomitant diseases. Nevertheless, administering IVIg at home began in France in the 1990s for reasons related to cost saving and to the patients' comfort and quality of life [12]. In this study, we present retrospective data on the use of IVIg (Tegeline) at high doses, administered in hospital and then at home in patients with AID, in order to assess the safety of Tegeline, as well as the conditions required for its administration at home.

## 2. Patients and Methods

### 2.1. Study Design

This was a multicenter, retrospective, observational, French study assessing and comparing the safety of Tegeline, an IVIg from LFB Biomedicaments, administered in hospital and then at home, in patients with AID. Twenty-two centers that manage patients with AID in France, including 17 neurology and 5 internal medicine departments, participated in the study.

To be eligible for the study, patients had to receive at least three consecutive cycles of Tegeline in hospital before starting treatment at home, at a dose between 1 (±0.1) and 2 g/kg/cycle, and then at least one cycle of Tegeline at home, at the same dose. Data for each patient were collected over the last 3 consecutive cycles of Tegeline administered in hospital, over all cycles administered at home, and up to 30 days after the last cycle of Tegeline administered at home, thus taking into account the time frame for the occurrence of possible AEs on IVIg [11, 13, 14]. Data taken from the medical files of patients treated with Tegeline in hospital and at home between 1 January 2000 and 1 April 2008 were collected from November 2008 to March 2009. All patients treated at home had a diary in which the home-care nurse recorded, for each cycle, the patient's vital signs, the dose of the administered IVIg, the infusion rate, and the nature of any AEs. In addition, data were collected within the study, using a questionnaire distributed to investigators, concerning the comfort or the improvement in comfort for patients treated at home.

The study was conducted in accordance with the regulations, that is, the French Code of Public Health, Good Clinical Practice, the principles set out in the Helsinki Declaration, and the Note for Guidance on IVIg (version dated 29 June 2000, available at the start of the study) based on EMA (European Medicines Agency) guidelines [15]. The latter stipulates that “data from at least 30 patients or 180 infusions” of IVIg are required to assess the safety of an IVIg in the context of a marketing authorization application for indications involving both substitution and immunomodulation. According to French clinical law, the protocol was not submitted to an ethics committee, being a retrospective study. All of the data collected underwent thorough quality control, that is, 100% verification of data source taken from the patient's medical files against data recorded in the patient's case report form.

All patients provided informed consent prior to collecting data.

### 2.2. Patient Profile

The included patients had to be at least 18 years old, suffering from well-diagnosed AID, requiring regular administrations of IVIg. They were treated with Tegeline at home for at least 1 cycle at a dose between 1 (±0.1) and 2 g/kg/cycle after having received treatment with Tegeline in hospital for 3 consecutive cycles at a dose between 1 (±0.1) and 2 g/kg/cycle without any SAE(s) during those cycles. The interval between cycles was left to the discretion of the investigators. Patients in whom Tegeline was administered alternately in hospital and at home were not included.

### 2.3. Study End Points

The primary objective of the study was to assess the safety of Tegeline administered at home at a dose between 1 (±0.1) and 2 g/kg/cycle in patients with AID. The primary end points to assess the objective were as follows:

- The number, nature, severity, seriousness, management, and outcome of AEs that occurred at home, as well as recurrence, if any, on rechallenge with Tegeline.
- Temporary or definitive discontinuation of administration of Tegeline at home with return to hospital due to AE(s).

The secondary objectives were (1) to compare the number, maximum severity, management, and outcome of AEs that occurred at home to those observed during the last 3 cycles of Tegeline administered in hospital; (2) to identify the criteria the physician considered necessary for the patient to be eligible for Tegeline administration at home at a dose between 1 (±0.1) and 2 g/kg/cycle; (3) to identify the risk factors that could potentially promote the occurrence of certain AEs at home; (4) to define the practical conditions required to ensure safe use of Tegeline at home; and (5) to assess the impact, for patients, of Tegeline administration at home as compared to hospital-based administration.

### 2.4. Statistical Analyses

The analysis set, or safety population, included all patients treated with Tegeline at home for at least one cycle at a dose between 1 (±0.1) and 2 g/kg/cycle after receiving treatment with Tegeline in hospital for 3 consecutive cycles.

The statistical analyses were essentially descriptive. Comparisons between hospital administration and at-home administration periods on different parameters were done using a Wilcoxon signed rank test and a two-sided significance level of 5%. Descriptive statistics involved patient numbers, number of missing data items, mean, standard deviation for the variable, standard deviation of the mean, maximum and minimum values, median and quartiles for quantitative parameters, and number and percentage for qualitative parameters.

The Cochrane-Mantel-Haenszel method was used to test the null hypothesis that there was no linear association between the variables analyzed in each of the strata (the strata comprised the patients). In order to adjust for a covariate, the sample was divided into strata. The two variables analyzed had to be at least ordinal. In the case of this present study, the correlation between the period in which the data were collected (i.e., hospital versus home) and the maximum severity of the AEs that occurred during each of the periods was tested.

### 2.5. Study Product

Tegeline is a freeze-dried IVIg, concentrated at 5% and stabilized with sucrose. It is manufactured by LFB Biomedicaments, a state-owned French pharmaceutical company. Tegeline is marketed primarily in France and also in other countries. In France, clinical trials have shown the efficacy and safety of Tegeline in some autoimmune diseases such MMN [16], CIDP [data ongoing for submission], Guillain-Barré syndrome (GBS), myasthenia gravis (MG) [17], ANCA-associated vasculitis [18], and neuropathy associated with primary Sjögren's syndrome [19]. Tegeline was the first immunoglobulin to obtain marketing authorization in France, for MMN in 2006 and for CIDP in 2009, in addition to classical indications, that is, PID, SID, ITP, GBS, bone-marrow transplant, and Kawasaki disease. Tegeline is also used in other autoimmune diseases including certain inflammatory myopathies according to the guidelines of the French Health Authorities for off-label IVIg use in France [20]. Moreover, as early as 2004, the French Health Authorities gave their approval for Tegeline to be administered at home in patients with immunodeficiencies according to data from a French clinical trial. Tegeline is used under the condition that the patient has previously been treated with Tegeline for at least 6 months in the hospital setting with no adverse reactions and that administration is to be initiated and monitored by a nurse or a person who has received specific training from the hospital team in charge of the patient [21].

## 3. Results

### 3.1. Patient Characteristics

All 46 eligible patients were included. The majority of patients (*n* = 38, 82.6%) were below 65 years of age. The male/female ratio was 1.5. Among the 46 included patients, thirty-three (71.7%) had immune-mediated neuropathy (MMN or CIDP). Among all of the treatments prescribed for AID, IVIg were reported to be the first-line treatment in 62% of the cases. In the remaining cases, they were primarily used after failure to obtain a partial or complete response with other therapeutic approaches such corticosteroids, immunosuppressants, and/or plasma exchange. The first IVIg cycle was used 7.9 months (mean) after the diagnosis of AID.

Corticosteroids and/or immunosuppressants were used in 71.7% and, among these patients, 50% were associated with Tegeline. Details of patient demographic data are shown in Table 1.

Among the 46 included patients, 28 patients (60.9%) had, according to the investigators, at least one risk factor for the occurrence of certain AEs with IVIg, that is, cardiovascular disease or cardiovascular risk factors (dyslipidemia, hypertension, and diabetes), chronic renal impairment, and so forth (Table 2). Weight > 100 kg and age > 65 years were not included as risk factors in this table. Mean blood creatinine values were normal at 75.3 *μ*mol/L (standard deviation (SD) 15.3) in hospital. On average, the patients had 1.17 risk factors (SD 1.37) with a median of 1.

In order to limit the occurrence of certain AEs at home, the investigators considered it necessary for patients to fulfil “eligibility criteria” before administering Tegeline at home. The most frequently reported “eligibility criteria” were “good understanding of the patient” (82.6%), “absence of acute or chronic renal impairment” (82.6% and 76.1%), “a well-diagnosed autoimmune condition requiring regular administration of IVIg” (80.4%), and “good safety profile of Tegeline in hospital” (78.3%). Other criteria are detailed for the same patients in Table 3.

### 3.2. Treatment

Mean follow-up of patients was 165.5 days (SD 129.1) in hospital, corresponding to 3 cycles of Tegeline according to the study design, and 282 days (SD 307.7) at home, corresponding to on average 7 cycles of Tegeline (1–40). During the follow-up period in hospital, 138 cycles of Tegeline were administered, at a mean dose of 1.6 g/kg/cycle (SD 0.4) over 2.83 days (SD 1.2), separated by 55.3 days (SD 45.4) on average. At home, 323 cycles of Tegeline were administered at a similar mean dose of 1.6 g/kg/cycle (SD 0.4), over 3.1 days (SD 1.2), every 50.2 days (SD 28.2). At least one precautionary measure to prevent certain AEs was reported in 31 of the 46 patients included in hospital. The measures were similar in hospital and at home for 23 patients and different for 6 patients (one fewer measure at home than in hospital for 3 patients and introduction of a new measure at home for the other 3). No precautionary measures were introduced at home in the 15 patients for whom no measures were taken in hospital. For 65.9% of Tegeline cycles realized in hospital (*n* = 91 on 138) and 50.8% at home (*n* = 164 on 323), at least one precautionary measure had been used. Details of precautionary measures are listed in Table 4.

### 3.3. Patient Management (Treatment at Home)

Almost all patients (97.8%) and their family member described as “spouse” or “child” (47.8%) had been “trained” on the modalities of at-home management while they were in hospital. For 43 of the 46 included patients (93.5%), management was entrusted to a home-care service provider and, for 2 patients (4.4%), to a home-hospitalization service provider. The information was missing for 1 patient. At home, Tegeline was administered in the majority of the cases using an infusion pump (60.9%, *N* = 28 patients) and, in 95.5% (*n* = 44 patients) of cases, in the presence of a family member. The vast majority of physicians (95.6%, *N* = 21 investigators) reported that they had frequent contacts with the service provider in charge of the patient.

### 3.4. Safety Results

The safety analyses revealed that, at home, 29 of the 46 patients included (63.0%) did not experience any AEs and that, among the 17 remaining patients, 45 AEs including serious adverse events (SAEs) in 3 patients were reported (Table 5). The severity of the 45 AEs (including SAEs) was assessed as mild in 30% of the cases, moderate in 62%, and severe in 8%. The AEs did not require any particular management in 35.6% of the cases. Symptomatic or curative treatment with or without temporary discontinuation of the infusion of Tegeline was required in 31.2% of the cases and a simple reduction in the infusion rate in 17.1% of the cases. The nonserious AEs reported at home were mainly headaches, hypertension, chills, fever, back pain, musculoskeletal pain, and nausea (Table 5). Among these 45 AEs reported at home, only 3, occurring in 3 different patients (6.5%), required hospitalization and were therefore considered as SAEs. The SAEs were one allergic reaction during the cycle, characterized by constrictive retrosternal chest pain, systolic hypotension, a general feeling of malaise, and somnolence that occurred at the 21st home cycle; one anaphylactoid reaction during the cycle, characterized by acute dyspnea and anaphylactic reaction that occurred between the 6th and 7th home cycles; and one erythematous rash occurring 6 weeks after the second cycle, characterized by erythema, muscle pain, diffuse joint pain, laboratory signs of inflammation, major asthenia on rising, mild fever, a feeling of swollen fingers, and increased gamma GT. The outcome was favorable for all SAEs in 3 patients. Administration of Tegeline was stopped but was resumed in hospital for all 3 patients (one week or about 2 months after the occurrence of SAEs) and then back home for 1 patient. All of the AEs that occurred at home resolved.

In hospital, 24 AEs (Table 5) occurred in 15 patients (32.6%). The severity of the AEs was assessed as mild in 33% of the cases, moderate in 63%, and severe in 4%. In 45.8% of the cases, the AEs did not require any particular management and, in 41.7%, simple symptomatic or curative treatment or simple reduction in the infusion rate was sufficient. All of the AEs that occurred in hospital resolved.

Among the 31 patients who had at least one risk factor, including weight > 100 kg and age > 65 years (31 patients, 3 of whom were aged above 65 as the only risk factor), only 17 patients experienced at least one AE. As for renal function, mean blood creatinine values were normal at 78.6 *μ*mol/L (±17.1) at home for 34 patients, compared to 75.3 *μ*mol/L (±15.3) in hospital for 44 patients (missing data for the remaining patients at home and in hospital).

### 3.5. Discontinuation of Tegeline at Home

Results of the statistical analysis showed that, among the 46 patients included and followed up during the observation period (January 2000 to April 2008), 45 patients continued their treatment with Tegeline at home or in hospital. One patient (2.2%) definitely discontinued administration of Tegeline at home due to disease worsening requiring a discontinuation of IVIg and second-line treatment. Thirty-nine patients (84.8%) were still receiving home treatment and 7 (15.2%) patients had definitely returned to hospital for their cycles (3 due to “patient's wish to return to hospital,” 1 due to “relocation abroad,” 1 due to “disease relapse” (exact cause, worsening?), and 2 due to “SAEs”). Four patients (8.7%) returned temporarily to hospital to receive their cycles of Tegeline (3 due to “placement/problem with implantable access port” and 1 due to “problem with infusion pump,” a problem that did not concern the patient directly) and then received Tegeline at home.

### 3.6. Comparison between Hospital and Home

The mean number of AEs per cycle was 0.17 (±0.33) in hospital and 0.23 (±0.47) at home. The mean number of AEs per month was 0.12 (±0.24) in hospital and 0.13 (±0.24) at home. The mean number of AEs per cycle and per month did not significantly differ, according to the Wilcoxon signed rank test, between hospital and home treatment (*p* = 0.605 and *p* = 0.452, resp.). The breakdown of the maximum severity of the AEs between hospital and home was similar: none (67.4% in hospital versus 63.0% at home), mild (13.0% versus 8.7%), moderate (17.4% versus 21.7%), and severe (2.2% versus 6.5%). Statistical analysis did not show any association between maximum intensity of the AEs and the place (hospital or home) of administration of Tegeline (*p* = 0.339). Statistical analysis did not show any significant association between the place of administration of Tegeline and the occurrence of AEs (*p* = 0.56). Because the number of AEs was low, the planned statistical analysis to assess the association between the presence of risk factors and the occurrence of certain AEs could not be performed.

### 3.7. Patient Satisfaction (Treatment at Home)

Data analyzed from the investigators' assessment revealed that, for 97.8% of the patients, home administration of cycles of Tegeline improved the patients' comfort in the range from “a little” to “enormously.” The improvement was primarily (84.4%) related to the fact that it was no longer necessary to travel from home to the hospital. Patients also expressed satisfaction for the following reasons: better morale in 51.1% of the cases, presence of their family during the cycles (42.2%), time saving (35.6%), more activities (33.3%) or maintained activity (24.4%), and better food (11.1%), among others.

## 4. Discussion

IVIg have been used at home in patients with immunodeficiencies since the end of the 1980s particularly in Northern Europe [7, 8] and in France since 2004 [21]. The use of IVIg at high doses in AID patients at home in France began in the middle of the 1990s [12]. There were several reasons, including cost saving, quality of life improvement, and time saving for patients.

Beyond the simple fact of showing that the results in France are similar to those reported in few earlier studies in the literature, the objective of this study was, first, to demonstrate that the AEs reported in this particular patient population do not differ in terms of frequency, nature, or severity depending on the place of administration of the IVIg (i.e., hospital versus home) and, secondly, to reassure hospital physicians who are reluctant today to undertake home administration of IVIg.

As early as 1994, the use of IVIg at high doses at home was authorized in the Netherlands for the treatment of MMN [22], under the condition that certain criteria had been met in advance. These included administering at least one cycle of IVIg (corresponding to a cumulative dose of 2 g/kg/cycle) in hospital, the presence of a nurse specialized in home management during the last cycle administered in hospital, prescription of an antiallergic reaction kit (epinephrine, prednisone, and an antihistamine), blood pressure monitoring, and verification of the possibility of a venous access port. Precautionary measures were taken in only 12 (23%) of the 52 patients in the study and consisted in the use of paracetamol (15%), of a nonsteroidal anti-inflammatory (4%), or of an antihistamine (4%). Only 2 SAEs were reported in 2 patients (4%), one characterized as a systemic reaction with somnolence and generalized rash and one pulmonary embolism due to an excessively high infusion rate at 11.5 mL/kg/hour. Both SAEs resolved and the cycles of IVIg at home were continued. Only 13% of patients experienced AEs during either the hospital or the home treatment period. The AEs were mild in severity, including headache, chills, or rash, and the frequency was similar (varying from a little less than 5% up to 59% for mild AEs and from 0 to 4.5% for SAEs) to that reported in cohorts (very widely studied) of patients with PID treated with IVIg [23, 24]. That study also demonstrated that the absentee rate at work was lower in patients managed at home than in those managed in hospital, further strengthening the medicoeconomic arguments in favor of home management.

In 2008, Rigas et al. performed a retrospective study to assess the safety of IVIg at home, involving 1085 cycles of high-dose IVIg administered only at home in patients with neuroimmunological disorders, primarily CIDP (63%), MG (11%), and polymyositis and dermatomyositis (8.5%), and other conditions including GBS (17.5%) [25]. Among the 70 patients included, 33% experienced a total of 51 AEs, that is, a rate of 4.7% for all cycles of IVIg administered. The authors explained the low rate of AEs to be due to the relatively low infusion rates for IVIg (compared to guidelines), as well as to the use of precautionary measures. The exact incidence of the precautionary measures could not however be investigated because of the retrospective nature of the study and the heterogeneous practices of physicians. Most of the AEs involved “headache” and “rash,” and 53% of all AEs occurred in 5 patients, indicating that certain patient profiles, that is, with risk factors of comorbidity and active neurological conditions, such as GBS or a flare-up of MG, are more likely to experience AEs [26]. This also underlines the importance of having eligibility criteria for administration of IVIg at home in order to limit the possible occurrence of AEs. No SAEs were reported. Moreover, analysis of 2 subgroups, that is, IVIg-naïve (*n* = 23) and previously treated with IVIg (*n* = 47), found that only 2 patients (9%) in the IVIg-naïve group experienced AEs (mild, transient rash and headache). The results of the study show that infusions of IVIg administered at home at high doses in patients with neuroimmunological disorders, under certain conditions, that is, chosen patient profile, low infusion rate, and precautionary measures, are well tolerated, even in patients who are naïve to treatment with IVIg.

The study performed by Souayah et al. in 2011 was the largest study to date in terms of the number of patients and analyzed cycles [26]. It included 4076 cycles of IVIg in 420 patients, divided into 334 patients with neuroimmunological disorders, mainly comprising CIDP (50%), receiving high-dose IVIg (Group 1), and 86 patients with immunodeficiency, receiving lower-dose IVIg (Group 2). The study demonstrated that there is a correlation between eligibility criteria for home treatment, that is, a particular patient profile, guidelines for proper use of IVIg in terms of administration conditions and implementation of precautionary measures, leading to a reduced risk of AEs during home administration of IVIg at high doses in patients with neuroimmunological disorders. A total of 90 patients, 72 patients (21.5%) in Group 1 and 18 patients (21%) in Group 2, experienced AEs, which were, in 95.5% of the cases, mild in severity and moderate in severity in 4.5% of cases. The overall frequency of AEs in the study (21.4%) was lower than those in the studies of Rigas et al. [25], Stangel et al. [27], Brannagan et al. [23], and Bertorini et al. [28], which were 33%, 57%, 59%, and 81%, respectively. As in the study by Rigas et al., no SAEs were reported [25]. It is important to point out that although the mean doses of IVIg administered and the mean age of patients in Group 1 were higher than those in Group 2, the incidence of AEs per cycle in Group 1 was not significantly different than that in Group 2 (2.28% versus 1.94%, *p* = 0.6). Precautionary measures were taken in a total of 276 patients (65.7%) during the study, and the incidence of AEs was significantly lower in this group of patients as compared to the group where precautionary measures were taken (18.4% versus 27.1%, *p* = 0.04). The lower incidence was even more marked in patients in Group 1 (18.2% versus 29.3%, *p* = 0.02). The precautionary measures mainly consisted in the use (alone or in association) of paracetamol, antihistamines, and corticosteroids.

In this retrospective study involving 46 patients who received a total of 461 cycles (138 in hospital and 323 at home) of Tegeline at high doses (1 ± 0.1 to 2 g/kg/cycle), we were able to assess the safety of Tegeline administered at home and to compare the safety of at-home versus hospital-based use of Tegeline. The occurrence of 45 AEs in 17 patients while receiving treatment at home compared to 24 AEs in 15 patients while receiving treatment in hospital is consistent with the literature data [23, 27, 28]. The nonserious AEs reported at home were those usually observed with Tegeline and other IVIg [13, 14]. The mean number of AEs per cycle and per month was not significantly different between hospital and home treatment (*p* = 0.605 and *p* = 0.452, resp.), and it can therefore be concluded that there is no significant link between the place of administration of Tegeline and the occurrence of AEs (*p* = 0.56). In addition to the analyses concerning the nature and the frequency of AEs, the present study, unlike the studies reported to date in the literature, also demonstrated that there is no link between the maximum severity of the AEs and the place of Tegeline administration (*p* = 0.339). Three SAEs in 3 patients were reported, which is consistent with the literature, where the frequency of SAEs varies from 3% to 16.6% [24, 27, 28]. None of the SAEs was life-threatening and although the patients initially had to return to hospital treatment, all three patients were able to continue treatment with Tegeline, 1 of them at home. It is important to point out that the causal relationship between Tegeline and the SAE “erythematous rash” seems questionable, given the time to onset of the SAE, that is, 6 weeks after administration of the last cycle of Tegeline, and considering that the mean plasma half-life of IVIg is around 23 days [14]. The same is true for the SAE described as “allergic reaction.” Indeed, the patient who experienced the SAE has been receiving Tegeline for more than 10 years, at the rate of one cycle every 6 weeks in hospital and then at home, and had progressively discontinued corticosteroid therapy, prescribed in the context of “polymyositis.” This treatment could be considered to have been a precautionary measure, and it is therefore surprising that, following the SAE, Tegeline was again reintroduced, with no particular precautionary measures, and no further AEs occurred.

The relatively good safety profile is however related to the fact that the patients in our study, along with those in earlier studies by Cats et al. [22], Souayah et al. [26], and Hachulla et al. [12], underwent a selection process with “eligibility criteria” prior to beginning at-home management. In this previous study, we identified seven eligibility criteria that would allow patients with AID to be considered for home treatment with IVIg. These were (1) the need for a defined diagnosis; (2) the presence of a rational physiopathological basis that could “legitimize” the use of IV immunoglobulin; (3) prescription by a senior hospital practitioner; (4) respect for the contraindications, that is, coronary artery disease, cardiac insufficiency or ischemic heart disease, recent stroke, nephropathy, uncontrolled hypertension, thrombosis of the infused veins, and hypersensitivity reaction after the first or second administration in hospital; (5) at least one hospital-based cycle of IVIg before starting infusions at home to assess safety; (6) mean infusion rate not exceeding 10 g over 2 hours; and (7) collaboration with a home-care service provider. In the present study, and unlike the study in 2002 [12], no eligibility (or inclusion) criteria for home treatment were considered. Indeed, we simply collected the main criteria that, according to the investigators, enabled them to define the patients profiles eligible for home treatment. Three of the four main criteria were found in both studies [12], and these were “absence of acute (82.6%) or chronic (76.1%) renal impairment,” “a well-diagnosed autoimmune condition requiring regular administration of intravenous immunoglobulins” (80.4%), and “good safety profile of Tegeline in hospital” (78.3%).

In addition to respect for these “eligibility criteria for home treatment,” the good safety profile of Tegeline is related to the use of precautionary measures, as shown in the study. Indeed, precautionary measures were taken in 67.4% of the patients, which is consistent with Souayah et al. [26] and Katzberg et al.'s [29] studies where measures were taken, respectively, in 65.7% and 100% of patients [26]. Finally, the good safety profile of Tegeline is also related to the good administration conditions of the product, which are defined as patients training (97.8%) and of those around them (47.8%) on the modalities of at-home management. In the large majority of cases (93.5%), management was performed by a home-care service provider. While collaboration with a service provider is not compulsory, in all cases, at-home administration of Tegeline, whether at high or low doses, should be initiated and monitored by a nurse or a person who has received specific training from the hospital team in charge of the patient. During the period of this study, 85% of the patients (*n* = 39) continued their cycles of Tegeline at home; 7 (15.2%) patients had definitively returned to hospital.

Our data, together with previous studies, clearly show the safety of home infusion of IVIg which has now become a routine practice [30]. Recently, subcutaneous self-infusion of immunoglobulins was considered in several immune-mediated neuromuscular diseases [31]. A large randomized controlled clinical trial for subcutaneous Ig infusion in CIDP (the PATH study) showed that both doses of SCIg IgPro20 (0.2 g/kg or 0.4 g/kg) were efficacious and well tolerated, suggesting that SCIg can be used as a maintenance treatment for CIDP and offering an interest for patients with poor venous access or severe treatment related fluctuations [32].

## 5. Conclusions

This study demonstrates the good safety profile of Tegeline administered at home at high doses, in patients with AID who are eligible to receive Tegeline at home and for whom the administration conditions had been assessed and validated by the hospital practitioner prior to the start of at-home management. After the end of this study, home-care-based intravenous immunoglobulins will become a common practice in France, especially in MMN and CIDP patients.

## Figures and Tables

**Table 1:** Patient characteristics (safety population, *N* = 46).

	Patient number (percentage)
Sex	
Female	18 (39.1%)
Male	28 (60.9%)
Age^*∗*^ (years)	
Mean (SD)	52.4 (13.24)
Median	53
(Minimum/maximum)	(25–79)
Weight (kg) in hospital versus at home	
Mean (SD)	74.3 (14.6) versus 74.5 (14.5)
Median	74.2 versus 75
(Minimum/maximum)	(49–127) versus (50–126)
Autoimmune diseases	
Motor multifocal neuropathy (MMN)	21 (45.7%)
Chronic inflammatory demyelinating polyneuropathy including Lewis-Sumner syndrome	12 (26.1%)
Polymyositis	3 (6.5%)
Dermatomyositis	3 (6.5%)
Body inclusion myositis	3 (6.5%)
Sclerosis with myopathy	1 (2.2%)
Gougerot-Sjogren syndrome with neurological forms	1 (2.2%)
Myasthenia gravis	1 (2.2%)
Cutaneous polyarteritis nodosa	1 (2.2%)
Family situation	
Living in a couple or in a family setting	41 (89.1%)
Living alone	5 (10.9%)
Professional activity at the time of administration of the cycles in hospital	
Professional activity	19 (42.2%)
Without professional activity	26 (57.8%)
(i) Retired person	17
(ii) Disabled person	6
(iii) Sick leave	1
(iv) Other	2

SD: standard deviation. ^*∗*^Calculated age = patient's age at the time of the last hospital-based cycle of Tegeline before starting treatment at home.

**Table 2:** Existing risk factors for the occurrence of certain adverse events (except age > 65 years or weight > 100 kg), according to the investigators' opinion.

Risk factors	Number of patients (%)
Total of patients with at least one risk factor	28/46 (60.9)
Dyslipidemia and/or hypercholesterolemia	14 (50.0)
Hypertension	11 (39.3)
Monoclonal gammopathy	6 (21.4)
Diabetes	4 (14.3)
Myocardial infarction	3 (10.7)
Migraine	2 (7.1)
Venous thromboembolic disease	2 (7.1)
Dyspnea	2 (7.1)
Chronic renal failure^*∗∗*^	1 (3.5)
Coronary insufficiency	1 (3.5)
Other risk factors^*∗*^	8 (3.5)

^*∗*^Risk factors mentioned by some investigators. ^*∗∗*^Moderate chronic renal impairment with blood creatinine at 103 *μ*mol/L during the Tegeline last cycles administered in hospital.

**Table 3:** Definition of patients' profile who could benefit from home-based intravenous immunoglobulin (Tegeline) by the investigators (safety population, *N* = 46).

	Total number of responses of investigators (%)
Good understanding of patient on the advantages and inconveniences of home-based IVIg	82.6
Absence of acute renal failure	82.6
Autoimmune disease well diagnosed and requiring administration of regular IVIg	80.4
Good safety of Tegeline at the hospital (during the 3 last consecutive cycles at the hospital before starting treatment at home)	78.3
Choice of patient	76.1
Absence of chronic renal failure	76.1
Minimum number of IVIg cycles realized at the hospital considered necessary, before starting treatment at home	76.1
Collaboration with a service provider, one visiting nurse, or home hospitalization	69.6
No venous problem	69.6
Prescription of Tegeline by a hospital doctor	65.2
Minimum/maximum rate of flow considered necessary for the hospital, before the administration of Tegeline at home	45.7
Absence of thrombosis in veins for the Tegeline infusion	39.1
Presence of a third person at home	15.2
Well-balanced hypertension	10.9
Well-balanced cardiopathy	8.7
Collaboration with a home-hospitalization service provider	6.5
Well-balanced diabetes	4.3
Well-balanced coronary insufficiency	4.3

**Table 4:** Precautionary measures used to prevent certain adverse events (in average rate, %) (analysis reduced to the patients having taken at least a precautionary measure, *N* = 31).

	Hospital	Home
	*N* of patients (%)	*N* (%)
Low-molecular-weight heparin or heparin	12 (38.7)	10 (32.2)
Corticosteroids	12 (38.7)	11 (35.5)
Hydratation before and/or after IVIg infusion (intravenous or oral)	17 (54.8)	13 (41.9)
Antihistamines	8 (25.8)	9 (29.0)
Analgesic	1 (3.2)	2 (6.4)
Drugs against hypertension	0 (0.0)	1 (3.2)

**Table 5:** Adverse events (AEs) in hospital and at home according to System Organ Class (SOC) and Preferred Term (PT) (MedDRA classification) (safety population, *N* = 46).

	Hospital		Home	
*System Organ Class*	*n* AE (%)^♠^	*N* patient (%)^◆^	*n* AE (%)^♠^	*N* patient (%)^◆^
Preferred Term				
*Total*	*24 (100)*	*15 (100)*	*45 (100)*	*17 (100)*
*Nervous system disorders*	*15 (62.5)*	*11 (73.3)*	*19 (42.2)*	*11 (64.7)*
Headache	15	11	18	10
Drowsiness^♣^	0	0	1	1
*Vascular disorders*	*2 (8.3)*	*2 (13.3)*	*9 (20.0)*	*5 (29.4)*
Hypertension	2	2	8	4
Hypotension^♣^	0	0	1	1
*General disorders and administration site condition*	*2 (8.3)*	*1 (6.7)*	*8 (17.8)*	*3 (17.6)*
Asthenia^♣^	0	0	1	1
Chest pain^♣^	0	0	1	1
Shivering	1	1	1	1
Hyperthermia	1	1	1	1
Malaise (feeling of faintness)^♣^	0	0	1	1
Peripheral edema^♣^	0	0	1	1
Pyrexia^♣^	0	0	1	1
Inflammation^♣^	0	0	1	1
*Musculoskeletal and connective tissue disorders*	*0 (0.0)*	*0 (0.0)*	*4 (8.9)*	*3 (17.6)*
Arthralgia^♣^	0	0	1	1
Back pain	0	0	1	1
Musculoskeletal pain	0	0	1	1
Myalgia^♣^	0	0	1	1
*Dysimmune system disorders*	*0 (0.0)*	*0 (0.0)*	*2 (4.4)*	*2 (11.8)*
Anaphylaxis reaction^♣^	0	0	1	1
Drug hypersensibility^♣^	0	0	1	1
*Intestinal disorders*	*4 (16.7)*	*2 (13.3)*	*1 (2.2)*	*1 (5.9)*
Nausea	3	2	1	1
Vomiting	1	1	0	0
*Chest, respiratory, and mediastinal disorders*	*0 (0.0)*	*0 (0.0)*	*1 (2.2)*	*1 (5.9)*
Acute dyspnea^♣^	0	0	1	1
*Skin and subcutaneous tissue disorders*	*1 (4.2)*	*1 (6.7)*	*1 (2.2)*	*1 (5.9)*
Eczema	1	1	0	0
Erythematous rash^♣^	0	0	1	1

*n AE (%)* ^♠^: *n* = number of AEs; (%) = *n*/total number of AEs (*n* = 24 at hospital; *n* = 45 at home); *N patient*^◆^: *N* = number of patients with an AE; (%) = *N*/number of patients with an AE (*n* = 15 at hospital; *n* = 17 at home). A patient could have many AEs during this study; ^♣^serious adverse event (*n* = 14).

## Abbreviations

AE(s): Adverse event(s)
AID: Autoimmune disease
ANCA: Antineutrophil cytoplasmic antibodies
CIDP: Chronic inflammatory demyelinating polyradiculoneuropathy
EMA: European Medicines Agency
GBS: Guillain-Barré syndrome
ITP: Immune thrombocytopenic purpura
IVIg: Intravenous immunoglobulins
MG: Myasthenia gravis
MMN: Multifocal motor neuropathy
PID: Primary immunodeficiency
SAE(s): Severe adverse event(s)
SD: Standard deviation
SID: Secondary immunodeficiency.
